# Pterostilbene mediates glial and immune responses to alleviate chronic intermittent hypoxia-induced oxidative stress in nerve cells

**DOI:** 10.1371/journal.pone.0286686

**Published:** 2023-06-02

**Authors:** Peijun Liu, Pan Zhou, Xinyue Zhang, Dong Zhao, Hao Chen, Ke Hu

**Affiliations:** Department of Respiratory and Critical Care Medicine, Renmin Hospital of Wuhan University, Wuhan, China; University of Rijeka Faculty of Medicine: Sveuciliste u Rijeci Medicinski fakultet, CROATIA

## Abstract

Chronic intermittent hypoxia (CIH) induces oxidative stress in the brain, causing sleep disorders. Herein, we investigated the role of pterostilbene (Pte) in CIH-mediated oxidative stress in the brain tissue. A CIH mouse model was constructed by alternately reducing and increasing oxygen concentration in a sealed box containing the mouse; brain tissue and serum were then collected after intragastric administration of Pte. Neurological function was evaluated through field experiments. The trajectory of the CIH mice to the central region initially decreased and then increased after Pte intervention. Pte increased the number of neuronal Nissl bodies in the hippocampus of CIH mice, upregulated the protein levels of Bcl-2, occludin, and ZO-1 as well as the mRNA and protein levels of cAMP-response element binding protein (CREB) and p-BDNF, and reduced the number of neuronal apoptotic cells, Bax protein levels, IBA-1, and GFAP levels. Simultaneously, Pte reversed the decreased levels of superoxide dismutase (SOD), glutathione peroxidase (GSH-PX), and BDNF and increased levels of malondialdehyde (MDA) in the serum of CIH mice. Pte increased Th2 cells, Treg cells, IL-4, IL-10, and TGF-β1 levels and decreased Th1 cells, Th17 cells, IFN-γ, IL-6, and IL- 17A levels in activated BV2 cells and hippocampus in CIH mice. The protein levels of p-ERK1/2, TLR4, p-p38, p-p65, and Bax, apoptosis rate, MDA concentration, Bcl-2 protein level, cell viability, and SOD and GSH-PX concentrations decreased after the activation of BV2 cells. Pte inhibited gliocytes from activating T-cell immune imbalance through p-ERK signaling to alleviate oxidative stress injury in nerve cells.

## Introduction

Obstructive sleep apnea syndrome (OSAS) can cause various diseases, including cardiovascular diseases and metabolic dysfunction. Neurocognitive impairment is a serious complication of OSAS [1, 2]. Chronic intermittent hypoxia (CIH) can cause nerve damage and cognitive dysfunction [3]. Clinical studies have reported hippocampal thinning in patients [4]. IL-1β, IL-6, TNF-α, and NF-κB are involved in the occurrence of neurocognitive dysfunction in patients with OSAS. Therefore, it is important to study the mechanisms of CIH in neurocognitive disorders and subsequently develop new targeted drugs.

Glial cells protect and support neurons and play an important role in CIH-induced neurocognitive disorders. When CIH-induced chronic inflammation occurs, glial cells are over-activated and rapidly migrate to damaged sites, transforming into a destructive M1 type, releasing large amounts of pro-inflammatory cytokines, affecting neuroplasticity and neurogenesis, and leading to cognitive deficits in mice [5, 6]. Microglia, another important regulator of innate and adaptive immune responses in the central nervous system, are critical for neuronal nutrition and the formation of the blood-brain barrier. Studies have shown that glial cells are over-activated during CIH; in this state, the expression of inflammatory factors and TLR2 in the hippocampus increases, resulting in hippocampal nerve injury and cognitive dysfunction. Seong et al. further confirmed that the TLR2 receptor can increase the proliferation, differentiation, and survival of neural stem cells after cerebral ischemic injury, thereby promoting the neurogenesis of hippocampal dentate gyrus neural stem cells [7]. TLR2 antagonists reduced hippocampal neuronal damage in a mouse sleep apnea model by inhibiting neuroinflammation and oxidative stress [8]. The activity of cAMP-response element binding protein (CREB) in neurons is related to a variety of intracellular processes, including proliferation, differentiation, survival, long-term synaptic enhancement, neurogenesis, and neuronal plasticity [9, 10]. Additionally, CREB activation promotes the expression of brain-derived neurotrophic factor (BDNF) [11]. BDNF is predominantly distributed in the central nervous system and is the most critical cytokine downstream of CREB. BDNF, which expression is the highest in the hippocampus, exerts a nutritional effect on the differentiation and proliferation of various types of neurons, plays a significant role in promoting the synthesis of neurotransmitters and neurotrophic factors, and is related to learning, memory, and cognitive processes [12, 13]. Therefore, preventing the effects of CIH on glial cell activity may be an important strategy to reverse CIH-induced neuropathology.

Pterostilbene (Pte) is a compound with a stilbene structure that is mainly found in blueberries. Studies have shown that resveratrol treatment can significantly improve cardiac function in CIH rats by mediating the PI3K/AKT/mTOR pathway and reducing myocardial hypertrophy, oxidative stress, and apoptosis [14]. Furthermore, it can abrogate hypoxia/reoxygenation-induced cardiomyocyte apoptosis in neonatal rats by activating the PI3K/AKT signaling pathway [15], indicating that both compounds mediate apoptosis through the PI3K/AKT pathway. Tanshinol treatment attenuates glutamate-induced oxidative stress injury in neuronal cells via the Nrf2 signaling pathway [16], whereas Pte inhibits oxidative stress and allergic airway inflammation through the AMPK/Sirt1 and Nrf2/HO-1 pathways [17]. In a previous study, Chen et al. confirmed that pyruvate activates immune cells [18]. CIH combined with a high salt diet (HSD) can increase the severity of hypertension by promoting the release of Th1-related cytokines (IFN-γ) and inhibiting anti-inflammatory cytokines (TGF-β1) [19]. Pte exhibits immunosuppressive activity by promoting the differentiation of CD4+ T cells into Tregs rather than into Th1 and Th17 cells [20]. Thus, Pte can mediate inflammation, oxidative stress, and immunity; however, the mechanism of CIH-induced neurological dysfunction has not yet been reported.

In summary, Pte alleviates CIH-induced neurological dysfunction by mediating inflammation, oxidative stress, and immunity. In the present study, a CIH mouse model was constructed using C57BL/6J mice treated with Pte. Microglia activation and brain tissue damage repair were evaluated, and the underlying mechanisms were studied from three perspectives: oxidative stress, inflammation, and immunity. Based on LPS+ TNF-γ-induced activated mouse microglia, we then evaluated the effects of Pte on the proliferation, apoptosis, oxidative stress, inflammation, and immunity of activated mouse microglia. Hence, the effect and specific mechanism of action of Pte on the neurological dysfunction caused by CIH in glial cells were explored, providing a new reference for CIH treatment.

## Material and methods

### Animals

Forty-eight male SPF grade C57BL/6 J mice, weighing 20–25 g, were obtained from the Hunan Lake JINGDA Company (Changsha, China). Mice were divided into four groups: normal oxygen supply (normal), normal oxygen supply + Pte intervention (normal + Pte), CIH, and CIH + Pte intervention (CIH + Pte). The normal + Pte and CIH + Pte groups were administered Pte by gavage once a day (30 mg/kg/day) [17]. For the normal and CIH groups, the same volume of normal saline was administered once a day. All mice were continuously gavage fed for four weeks; after collecting the blood from the eyeballs of all mice, the mice were euthanized by decapitation, serum was collected, and brain tissue were collected and frozen in formalin solution for storage. All animal procedures in this study were performed in accordance with the Regulations of Experimental Animal Administration issued by the State Committee of Science and Technology of the People’s Republic of China, with the approval of the Ethics Committee of Renmin Hospital of Wuhan University (IACUC Issue No: 20220501A).

### CIH modeling

Mice in the CIH group were placed in a sealed box for hypoxia-oxygen recovery. Nitrogen and oxygen were injected into the box, and oxygen was withdrawn such that the oxygen concentration was reduced from 21% to 8% and maintained for 10 s. The oxygen concentration was then restored to 21% and maintained for 50 s. This procedure was repeated daily from 9 a.m. to 5 p.m. for four consecutive weeks.

### Open field test

At the end of the experimental intervention (the fifth week), the mice were placed in the open field laboratory for 30 min in advance, and the movement trajectory of each mouse within 5 min and residence time in the central area were recorded using Supermaze software.

### Cell culture

BV2 mouse microglia were purchased from Procell (Wuhan, China) and cultured in MEM (containing NEAA + 10% FBS + 1% P/S) in a 37°C, 5% CO_2_ environment. The cells were divided into three groups: control (0), LPS + IFN-γ induced (LPS + IFN-γ), and LPS + IFN-γ + Pte induced (LPS + IFN-γ + Pte). Pte (10 μM) was pretreated for 30 min; we then added LPS (100 ng/mL) and IFN-γ (5 ng/mL) intervene 24 h [17, 21].

### Western blot

Proteins were extracted from the hippocampal tissue and BV2 cells. Lysis buffer and kits were used for quantification (Solarbio, Beijing, China). Proteins were separated using sodium dodecyl sulfate-polyacrylamide gel electrophoresis and transferred onto polyvinylidene difluoride (PVDF) (Millipore, US). Blocking was performed with 5% skimmed milk, after which the PVDF was incubated for 12 h with primary antibodies against Bax (Bioswamp, Wuhan, China), Bcl-2 (Bioswamp), CREB (Bioswamp), p-CREB (Bioswamp), BDNF (Bioswamp), ERK1/2 (Bioswamp), p-ERK1/2 (CST), p38MAPK (Bioswamp), p-p38MAPK (Abcam), TLR4 (Bioswamp), NF-κB p65 (Bioswamp), p-NF-κB p65 (Abcam), and GAPDH (Bioswamp). The membranes were incubated with a secondary antibody (Bioswamp) for 2 h, and the protein bands were scanned.

### RT-qPCR

Total RNA was extracted from mouse hippocampal tissue and BV2 cells using TRIzol reagent, and RNA integrity was confirmed using agarose gel electrophoresis. Reverse transcription of cDNA from mRNA was performed using a reverse transcription kit (TAKARA, Dalian, China). RT-qPCR was then performed using the SYBR Green method (KAPA Biosystems). The primer sequences are listed in Table 1. The relative gene expression level was analyzed using the 2^-ΔΔCt^ method.

**Table 1:** Primer sequences and sizes.

Primers	Sequence	Size
CREB-F	AGTGCCCAGCAACCAAG	113
CREB-R	GGGAGGACGCCATAACA	
BDNF-F	TTATTTCATACTTCGGTTGCA	166
BDNF-R	GTGTCAGCCAGTGATGTCG	
GAPDH-F	CCTTCCGTGTTCCTAC	152
GAPDH-R	GACAACCTGGTCCTCA	

### Enzyme-linked immunosorbent assay (ELISA)

For the ELISA test, 100 μL of standard was added to 100 μL standard diluent and mixed well, and 100 μL of the above mixture was then added to 100 μL standard diluent again and mixed well; this was repeated six times. The serum and cell samples, BDNF, IFN-γ, IL-6, IL-17A, IL-4, IL-10, and TGF-β1 antibodies were then added into the blank well of the ELISA plate and mixed before adding the HRP-conjugate reagent; they were incubated at 37°C for 30 min. The liquid was then discarded from the plate, which was then dried by rotation, and washed for 30 s; this procedure was repeated five times. A chromogenic agent was then added, incubated at 37°C in darkness for 10 min, and the termination solution was added. Optical density value was measured at a 450 nm wavelength. All the reagents were obtained from an ELISA kit (Bioswamp).

### Biochemical analysis

The serum samples and BV2 cells were analyzed using a Shenzhen Mindray BS-420 automatic biochemical analyzer, and SOD (U/mg protein), MDA (nmol/mg protein), and GSH-PX (U/mg protein) concentrations were determined using a Shenzhen Mairui (Shenzhen, China) matching biochemical kit.

### Immune cell detection

Three groups of BV2 microglia with different treatments (control (0), LPS + IFN-γ, and LPS + IFN-γ + Pte) were co-cultured with CD4+ T cells, and the ratio of Th1/Th2 and Treg/Th17 in cellular CD4+ T cells was detected using flow cytometry. CD4-FITC (Invitrogen), CD25-APC (Invitrogen), and IL-17 (PE) were used to detect Tregs. CD4-FITC and IFN-γ (PE) were used to detect Th1 cells. CD4-FITC and IL4 (PE) were used to detect Th2 cells. CD4-FITC and IL-17 (PE) were used to detect Th17 cells.

### Cell Counting Kit-8 (CCK8)

Cells were treated according to the parameters for the different groups for 24 h. CCK8 solution (Solarbio) was added to the culture for 4 h. The absorbance of each well was measured at 450 nm, and the cell survival rate was calculated.

### Flow cytometric analysis of apoptosis

The cells were stained with 10 μL of Annexin V-fluorescein isothiocyanate (BD, US) and 10 μL of propidium iodide (BD, US) in the dark for 30 min. After adding 300 μL of PBS, flow cytometry was performed using a NovoCyte apparatus (ACEA Biosciences, China).

### Hematoxylin eosin staining

Paraffin-embedded hippocampal tissue was sectioned, and the sections were stained with hematoxylin (Bioswamp). The sections were then immersed in 1% hydrochloric acid alcohol for 3 s, washed with water for 2 s, immersed in a bluing solution for 10 s, washed with running water for 30 s, stained with 0.5% eosin solution (Bioswamp) for 3 min, washed for 2 s with distilled water, and immersed in increasing concentrations of ethanol. After one dip in anhydrous ethanol and two washes with xylene, the sections were sealed with neutral balsam gum and observed under an optical microscope.

### Immunofluorescence

The protein expression of IBA-1 and GFAP in the hippocampal tissue was examined using immunohistochemistry. After embedding and slicing, the tissue sections were dewaxed and hydrated before antigen retrieval using citrate buffer. The sections were then blocked with 3% H_2_O_2_ for 15 min and immersed in 10% goat serum for 20 min. They were then incubated with a primary antibody (Bioswamp) overnight and the Max Vision^TM^ HRP-polymer anti-mouse/rabbit secondary antibody (Fuzhou Maixin Biotechnology Development Co. LTD) for 45 min at room temperature. An anti-fluorescence attenuation sealing tablet was then added (including DAPI, Solarbio). The sections were observed under a fluorescence microscope (Leica, Germany).

### Immunohistochemistry staining

The protein expression of occludin and ZO-1 in hippocampal tissue was examined using immunohistochemistry. After embedding and slicing, the tissue sections were dewaxed and hydrated before antigen retrieval using citrate buffer. The sections were blocked with 3% H_2_O_2_ for 15 min and immersed in 10% goat serum for 20 min. They were then incubated with the primary antibody (Bioswamp) overnight and Max Vision ^TM^ HRP-Polymer anti-Mouse/Rabbit IHC secondary antibody (Fuzhou Maixin Biotechnology Development Co., LTD) for 45 min at room temperature. The samples were visualized using diaminobenzidine (Solarbio) and counterstained with hematoxylin (Solarbio). The sections were observed under a microscope (Leica, Germany).

### TUNEL staining

Mouse hippocampal tissue was immersed in wax, embedded, frozen, and cut into 3 μm sections. The sections were attached to glass slides and baked at 65°C. The glass slides were immersed in xylene for 25 min and then immersed in 100–75% gradient alcohol for 3 min. Proteinase K (Solarbio) was added at 37°C. After 15 min, the TUNEL mixture (Roche, Shanghai, China) was added and incubated for 60 min. After transformation, DAB was added; the glass slides were cleaned and stained with hematoxylin, and neutral resin was dropped to capture images.

### Nissl staining

Hippocampal tissue sections were routinely dewaxed in water. Nissl staining solution (Servicebio, Wuhan, China) was added to dye the sample for 2–5 min; slides were then dried at 65°C, and neutral resin was dropped to take photos.

### Statistical analysis

Statistical analyses were performed using SPSS2.0 software. All data are presented as the mean ± standard deviation (SD). Student’s t-test was used for comparisons between two groups. Statistical significance was set at P < 0.05. * P<0.05 vs. normal group. # P<0.05 vs. CIH group.

## Results

### Role of Pte in CIH-induced nerve injury and glial cell activation in mice

**Fig 1:**
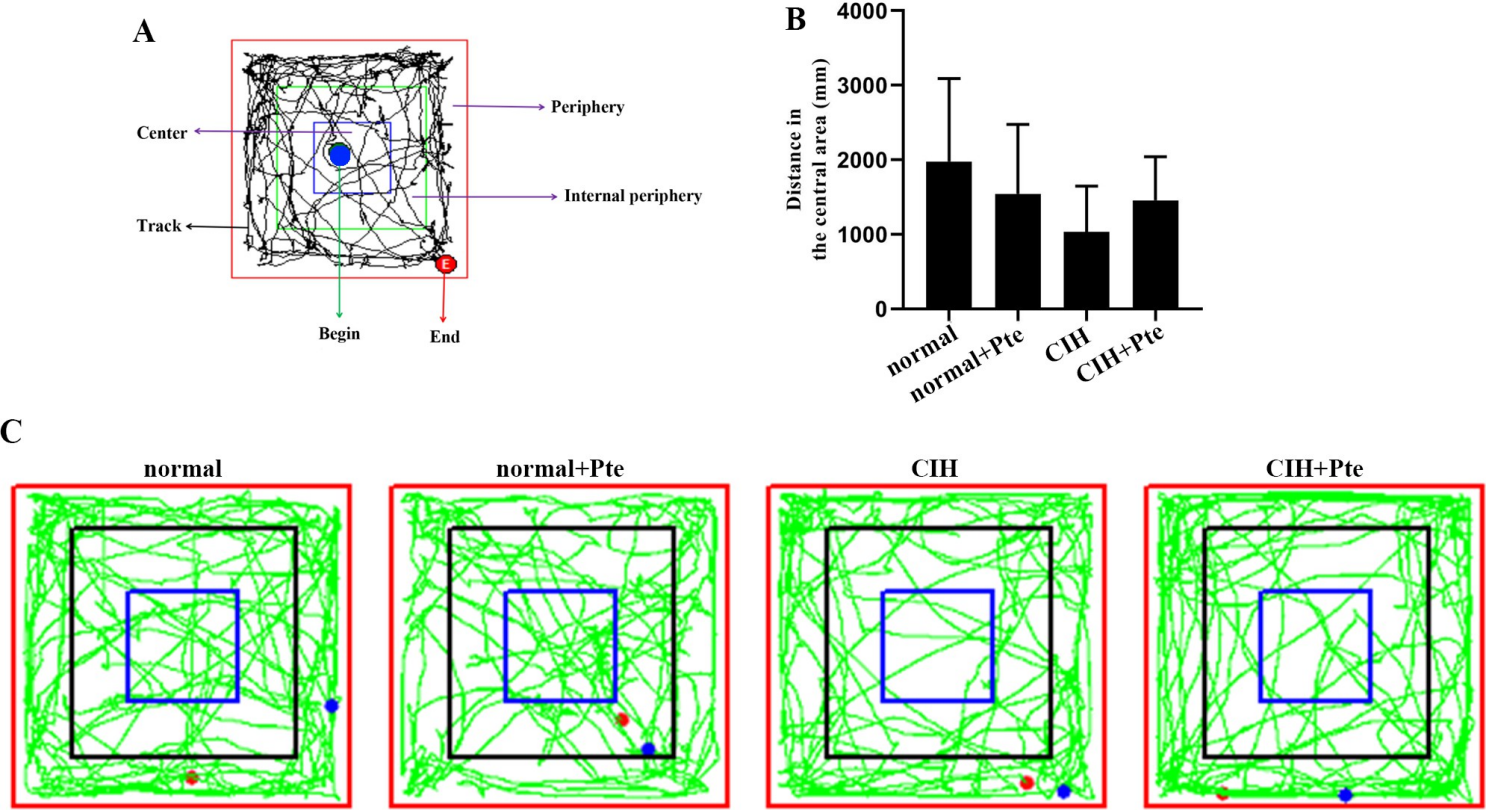
Open field test.

**Fig 2:**
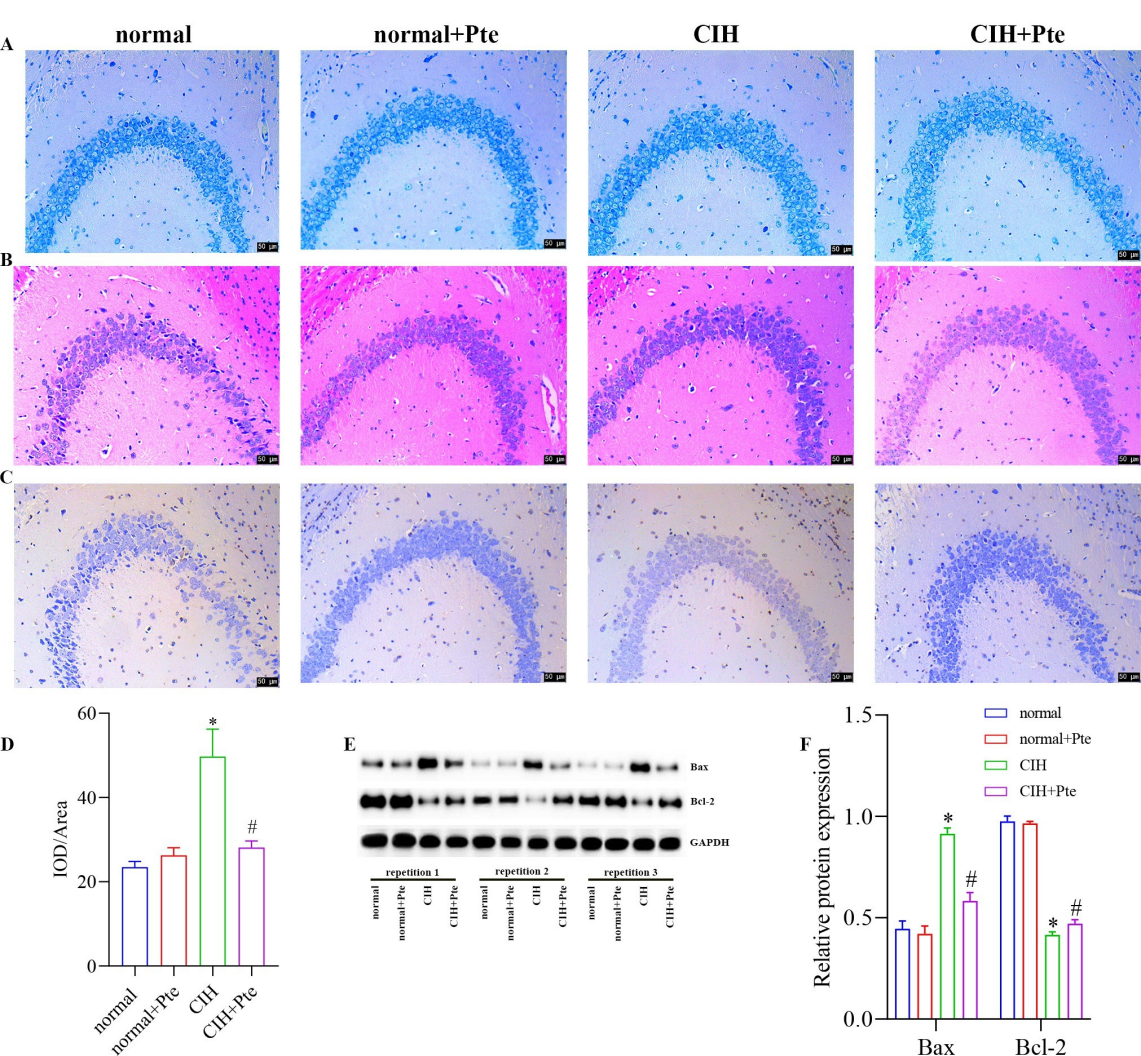
Role of Pte in CIH-induced nerve injury and glial cell activation in mice. (A) Nissl staining. (B) Hematoxylin eosin staining. (C, D) TUNEL staining. (E, F) Protein levels of Bax and Bcl-2 were detected using western blot. * P < 0.05 vs normal group. # P < 0.05 vs CIH group. Scale bar, 50 μm.

**Fig 3:**
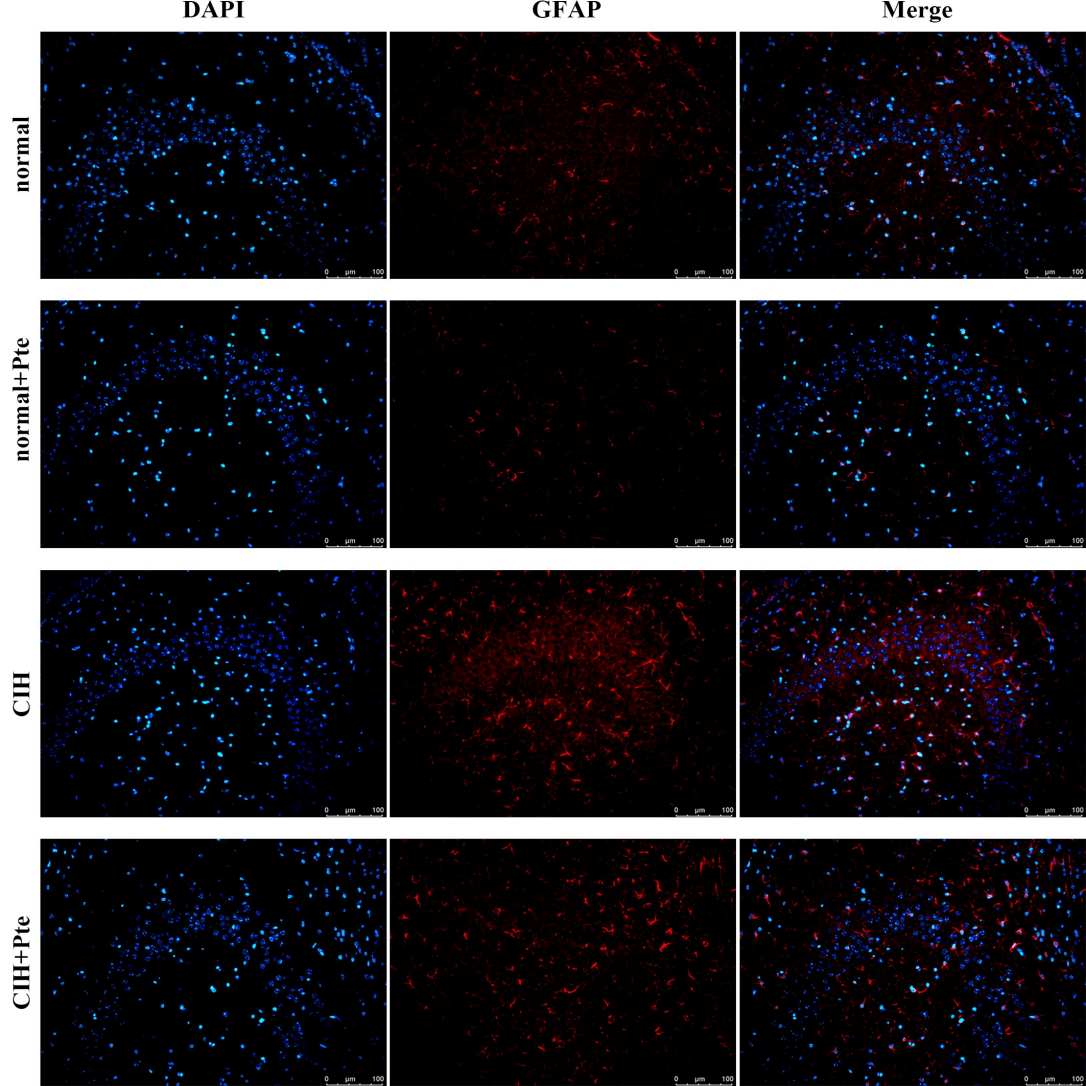
Protein expression of GFAP in brain tissues examined using immunohistochemistry. Scale bar, 100 μm.

**Fig 4:**
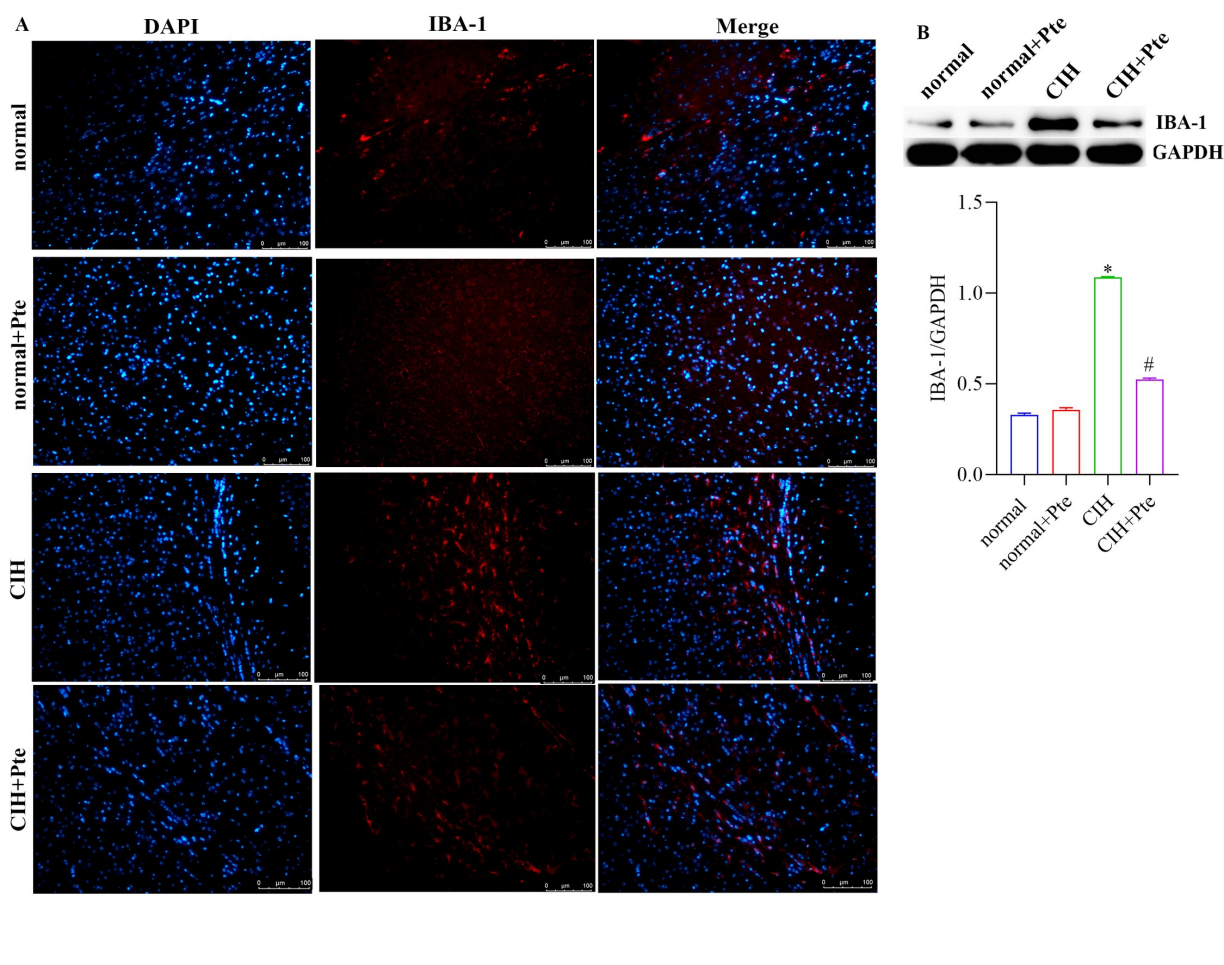
Role of Pte in glial cell activation in mice. (A) Protein expression of IBA-1 in brain tissues was examined using immunohistochemistry. Scale bar, 100 μm. (B) Detection of IBA-1 protein expression using western blot.

The open field test revealed that mice in the CIH group had the shortest trajectories in the central region, whereas mice in the CIH + Pte group had increased trajectories in the central region compared with those of mice in the CIH group (Fig 1). Compared to the normal group, the CIH group showed decreased Nis-type bodies, disordered cell arrangement, ruptured nerve membranes, increased number of apoptotic cells and Bax protein levels, and decreased Bcl-2 protein levels in the hippocampal tissue. Compared to those of the CIH group, the number of Nissl bodies in the hippocampus of the CIH + Pte group was increased, the level of Bax protein with intact neural cell morphology was decreased, and the level of Bcl-2 protein was increased (Fig 2 and S1 Fig). GFAP and IBA-1 proteins were detected using immunofluorescence. As shown in Figs 3 and 4, compared with those of mice in the normal group, the protein fluorescence levels of GFAP and IBA-1 (S2 Fig) in the hippocampus of mice in the CIH group were increased. Furthermore, the protein fluorescence levels of GFAP and IBA-1 in the hippocampus of mice in the CIH + Pte group were decreased compared with those of mice in the CIH group. These results indicated that CIH induced brain nerve injury and glial cell activation in mice.

### Role of Pte in CIH-induced oxidative stress in mice

**Fig 5:**
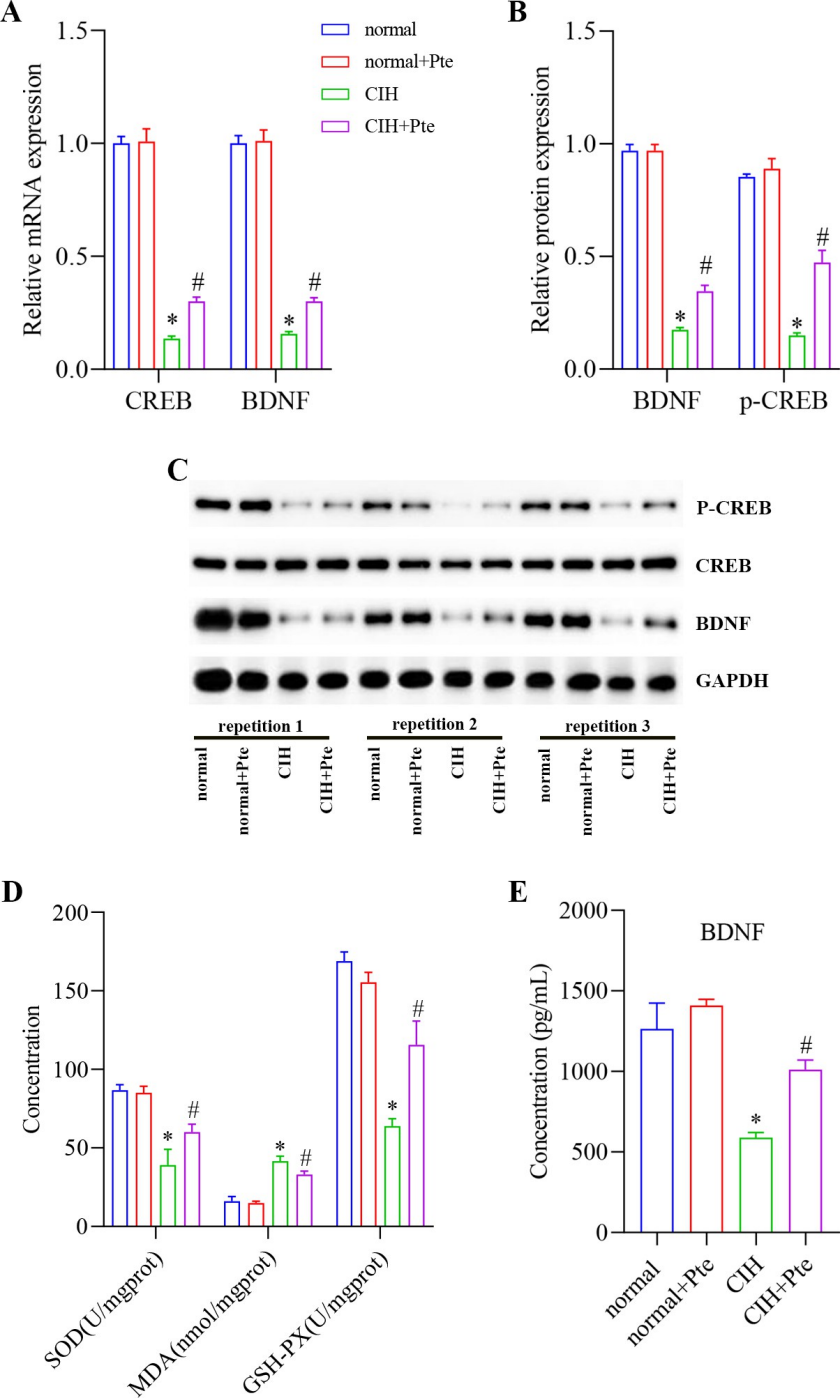
Role of Pte in CIH-induced oxidative stress in mice. (A) mRNA levels of CREB and BNDF detected using RT-qPCR. (B, C) Protein levels of p-CREB and BNDF detected using western blot. (D, E) Concentrations of MDA, BNDF, SOD, and GSH-PX detected using ELISA and biochemical analysis. * P < 0.05 vs. normal group. # P < 0.05 vs. CIH group.

**Fig 6:**
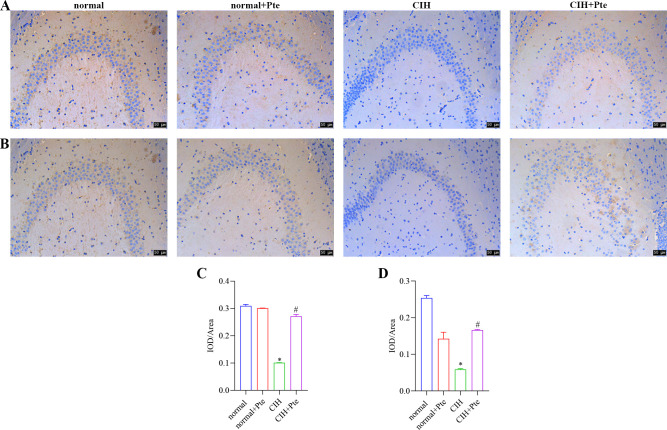
Protein expression of occludin and ZO-1 in brain tissues examined using immunohistochemistry. (A, C) Protein expression of occludin in brain tissues examined using immunohistochemistry. (B, D) Protein expression of ZO-1 in brain tissues examined using immunohistochemistry. *P < 0.05 vs. normal group. # P< 0.05 vs. CIH group. Scale bar, 50 μm.

In the hippocampal tissue and serum of mice, the mRNA and protein levels of p-CREB and BNDF were lower in the CIH group than those in the normal group and higher in the CIH + Pte group than those in the CIH group (Fig 5A–5C and S3 Fig). Compared to those in the normal group, the concentrations of BNDF, SOD, and GSH-PX in the CIH group decreased, whereas the concentration of MDA increased (Fig 5D and 5E). In addition, occludin and ZO-1 protein levels, which are associated with the blood-brain barrier, were reduced. Compared to those in the CIH group, the concentrations of BNDF, SOD, and GSH-PX increased; the concentration of MDA decreased; and the occludin and ZO-1 protein levels increased in the CIH + Pte group (Fig 6).

### Role of Pte in CIH-induced immune imbalance in mice

**Fig 7:**
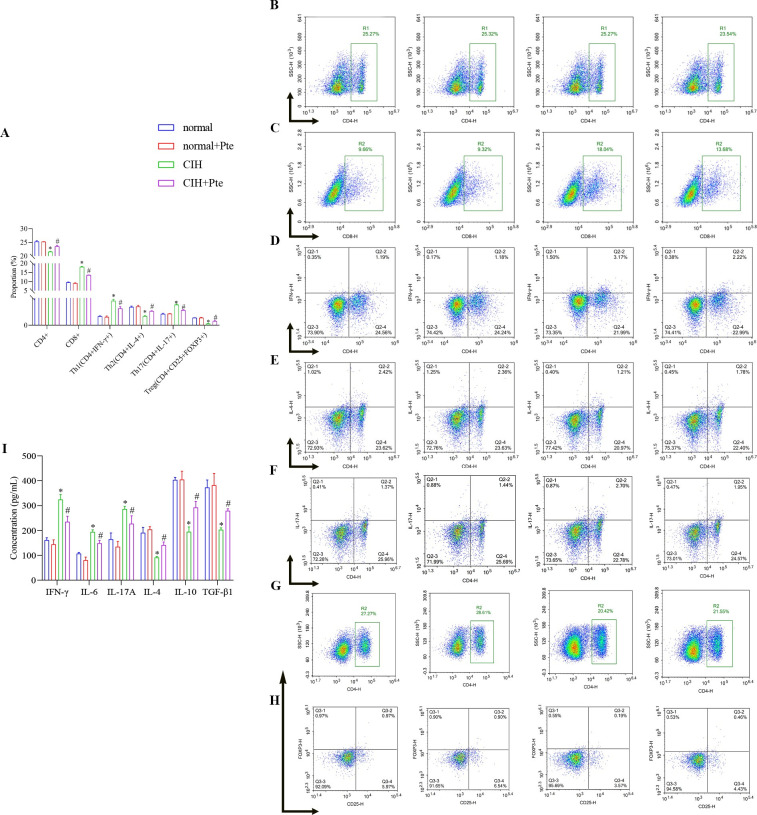
Role of Pte in CIH-induced immune imbalance in mice. (A–H) Proportions of Th1, Th2, Th17, and Treg cells in the peripheral blood was analyzed using flow cytometry. (I) Concentrations of IFN-γ, IL-6, IL-17A, IL-4, IL-10, and TGF-β1 were detected using ELISA. *P < 0.05 vs. normal group. # P < 0.05 vs. CIH group.

As shown in Fig 7, compared to those in the normal group, the ratio of Th2 and Treg cells decreased, whereas the ratio of Th1 and Th17 cells increased in the CIH group. Corresponding to this result, the concentrations of IFN-γ, IL-6, and IL-17A were increased, whereas the concentrations of IL-4, IL-10, and TGF-β1 were decreased. Compared with those in the CIH group, the ratios of Th2 and Th17 cells were increased; the ratios of Th1 and Treg cells were decreased; the concentrations of IFN-γ, IL-6, and IL-17A were decreased; and the concentrations of IL-4, IL-10, and TGF-β1 were increased in the CIH + Pte group (Fig 7 and S1 File).

### Role of Pte in BV2-activated cells

**Fig 8:**
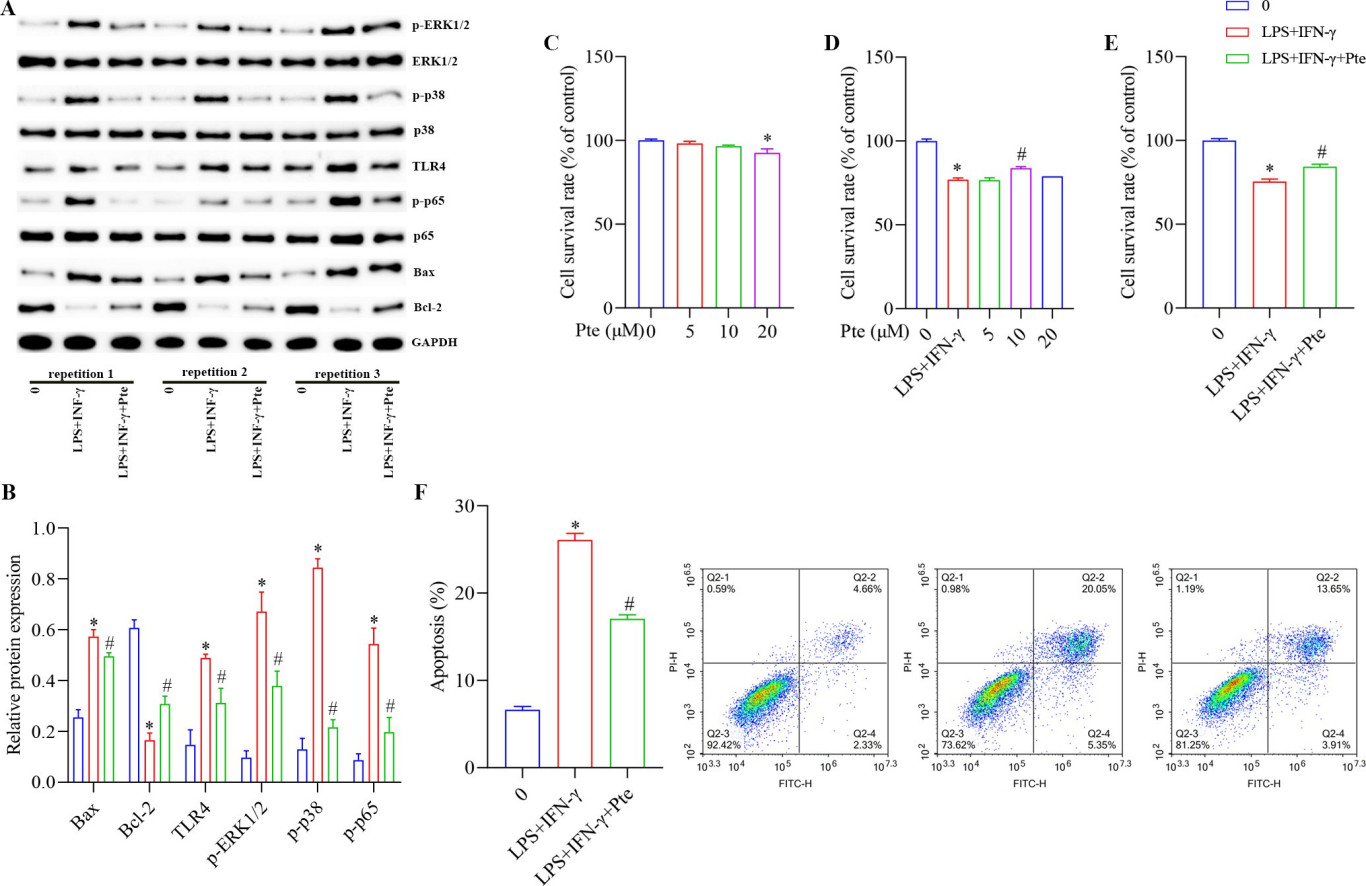
Role of Pte in BV2-activated cells. (A, B) Protein levels of Bax, Bcl-2, ERK1/2, p-ERK1/2, p38, p-p38, TLR4, p65, and p-p65 detected using western blot. (C-E) CCK8 to detect cell viability. *P < 0.05 vs. group 0, # P < 0.05 vs. LPS + IFN-γ. (F) Flow cytometric analysis of apoptosis. *P < 0.05 vs. group 0. # P < 0.05 vs. LPS + IFN-γ group.

**Fig 9:**
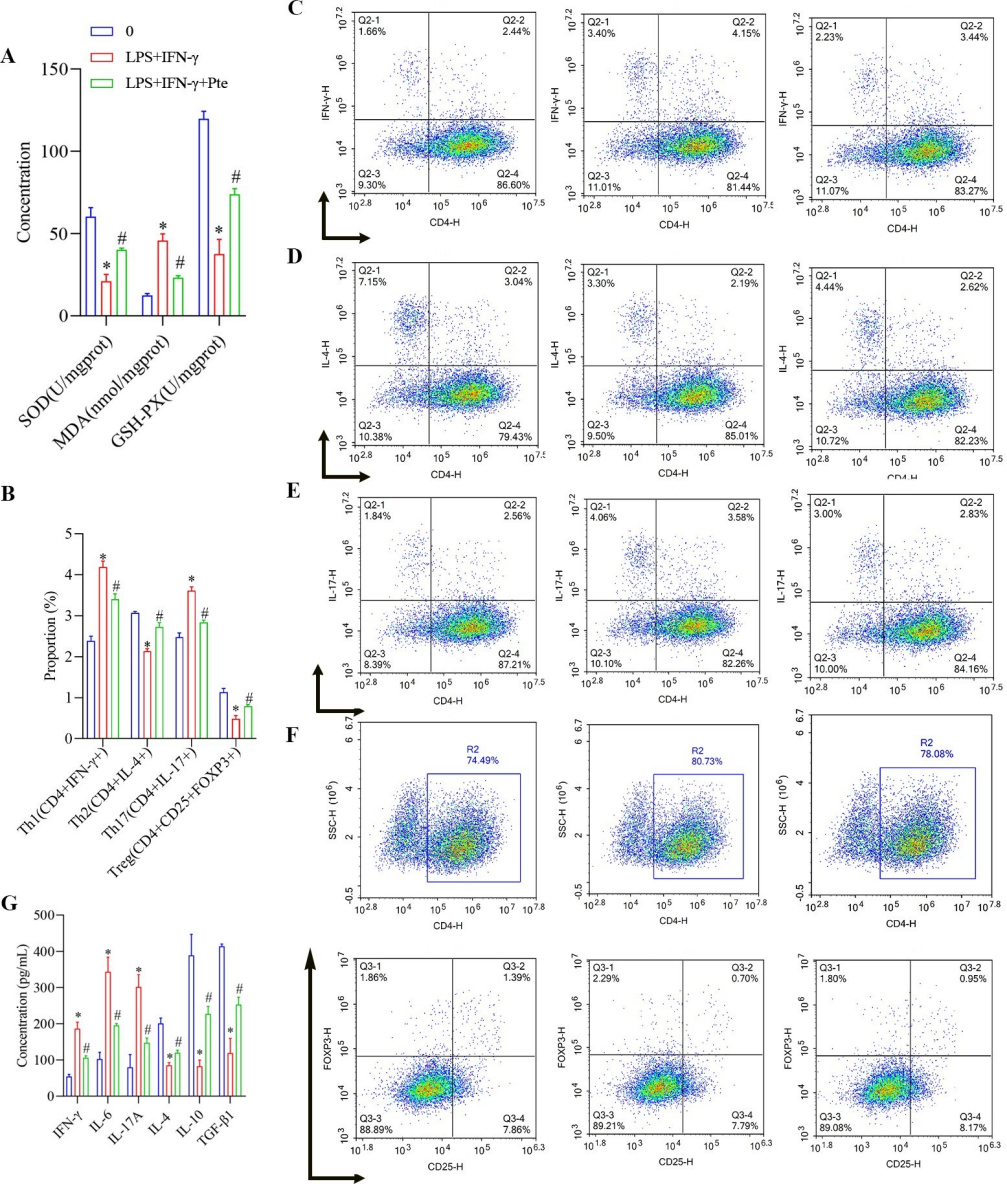
Role of Pte in BV2-activated induction of immune imbalance in cells. (A) Concentrations of MDA, SOD, and GSH-PX were detected using biochemical analysis. (B-F) Proportions of Th1, Th2, Th17, and Treg cells in BV2 were analyzed using flow cytometry. (G) Concentrations of IFN-γ, IL-6, IL-17A, IL-4, IL-10, and TGF-β1 were detected using ELISA. *P < 0.05 vs. group 0. # P < 0.05 vs. LPS + IFN-γ group.

As shown in Fig 8 and S4 Fig, compared with those in group 0, the protein levels of Bax, p-CREB, p-ERK1/2, p-p38, TLR4, and p-p65 were increased, the protein levels of Bcl-2 were decreased, and the apoptosis rate was increased in the LPS + IFN-γ group. The survival rate of BV2-activated cells was significantly reduced by 20 μM Pte. Compared with those in the LPS + IFN-γ group, the protein levels of Bax, p-CREB, p-ERK1/2, p-p38, TLR4, and p-p65 were decreased; the protein levels of Bcl-2 were increased; and the apoptosis rate was decreased in the LPS + IFN-γ + Pte group. The survival rate of BV2-activated cells also increased. In addition, compared with those in the group 0, SOD and GSH-PX concentrations were decreased; MDA concentrations were increased; Th2 and Treg cell ratios were decreased; Th1 and Th17 cells ratios were increased; IFN-γ, IL-6, and IL-17A concentrations were increased; and IL-4, IL-10, and TGF-β1 were decreased in the LPS + IFN-γ + Pte group. Compared with those in the LPS + IFN-γ group, SOD and GSH-PX concentrations were increased; MDA concentrations were decreased; Th2 and Treg cells ratios were increased; Th1 and Th17 cell ratios were decreased; the concentrations of IFN-γ, IL-6, and IL-17A decreased; and the concentrations of IL-4, IL-10, and TGF-β1 increased in the LPS + IFN-γ + Pte group (Fig 9, S2 File).

## Discussion

OSAS is a common sleep disorder, for which CIH is a common symptom. Patients with OSAS and CIH often show high levels of oxidative stress [22, 23]. Previous studies have demonstrated that CIH can induce oxidative stress in the brain tissue involved in spatial memory function [24]. Pte also protects against oxidative stress injury in microglia [25], suggesting that Pte is strongly correlated with neuronal oxidative stress. Thus, proposing that Pte could be used to treat CIH is reasonable. Unfortunately, there are only few reports on the relationship between Pte and CIH. The effect and mechanism of action of Pte in improving oxidative stress in the brain tissue caused by CIH remain unclear and have not yet been reported.

In the present study, we explored the effects of a normal oxygen environment and hypoxic deactivation on the CIH mice hippocampi. The number of Nissl bodies in the hippocampal neurons decreased; apoptosis increased; and the protein levels of p-CREB, BDNF, IBA-1, GFAP, occludin, and ZO-1 increased significantly. Furthermore, the proportions of Th1/Th2 and Th17/Treg immune cells and their corresponding cytokines increased significantly. Our results indicated that hypoxia induced oxidative stress and nerve cell damage in CIH mice brain tissue as well as changes in the proportion of immune cells. Shen et al. [26] found that the expression levels of p-CREB, BDNF, and p-ERK1/2 in CIH mice increased after the injection of PNU-282987. Similarly, Wang et al. [27] suggested that CIH-induced neurocognitive dysfunction is related to the dephosphorylation of ERK1/2 and CREB. In this study, p-CREB and BDNF were significantly increased in the hippocampus after Pte treatment, whereas p-ERK1/2 was significantly increased in BV2 cells, indicating that Pte alleviated hippocampal nerve injury by regulating p-CREB and BDNF. Previous studies have shown that oxidative stress injury is associated with cognitive impairment following CIH [28, 29]. In the present study, we demonstrated that CIH mice exhibited significant anxiety, with disordered cell arrangements, ruptured neuromembranes, and significantly damaged nerves. After treatment with Pte, anxiety in the mice was significantly reduced, and the morphology of nerve cells was significantly improved. Therefore, we suggested that Pte could ameliorate nerve damage by alleviating anxiety and cognitive dysfunction in mice.

Hippocampal cells are dominated by neurons, whereas microglia are the main immune and inflammatory cells in the brain that exert multiple functions, including antigen presentation and production of pro-inflammatory or anti-inflammatory factors, etc [30–32]. Several studies have shown that microglial activation plays an important role in chronic CIH [33–35]. In this study, hippocampal tissue and nerve cells were damaged because immunofluorescence and western blot experiments showed the activation of IBA-1 proteins, which was why activated BV2 cells were used in the cell experiments. CIH is a sign of sleep disorders, which is a common early symptom of neurodegenerative diseases. Lin et al. [36] found that increased number of microglia were the cause of neuropathology in CIH. This verified that Pte might reduce CIH nerve injury by regulating microglial activation.

Several studies have found that activation of microglia may be a potential target to limit brain inflammation and oxidative stress after sleep deprivation [37]. In this study, the activation of microglia induced cellular oxidative stress. Since Pte reduced not only the activation of microglia but also cellular oxidative stress, the levels of SOD and GSH-PX increased, whereas MDA levels decreased after Pte intervention in the hippocampus and BV2 cell model. Xue et al. [37] found that microglial activation was accompanied by oxidative stress in sleep-deprived mice. Furthermore, Wu et al. [34] showed that the levels of oxidative stress (MDA and SOD) were promoted in microglial activation induced by CIH. Conversely, the reduction of ROS and MDA production in microglia partially reversed the oxidative stress.

In the present study, we found that an immune imbalance existed in both the hippocampus and the constructed BV2 model. The increased proportion of Th1/Th2 and Th17/Treg immune cells found in the BV2 cell model explained the nerve damage in the hippocampus. Jiang et al. [38] found that CIH changed the proportion of Th17 cells and Treg cells via the regulation of HIF1α and mTOR signaling. Studies on Pte and T-cell immune regulation have only been reported in colitis [20]. Therefore, our novel findings may provide new insights into the ability of Pte to reduce the imbalance between Th1/Th2 and Treg/Th17 immune cells and hinder the activation of microglia, as we found that Pte might affect the activation state of microglia by regulating the immune response and alleviating nerve injury in CIH mice.

The imbalance of immune cells and induction of neurological impairment in the central nervous system are associated with both occludin and ZO-1 proteins [39]. Previous studies have shown that mice fed a high-fat diet exhibit immune cell infiltration, decreased expression of ZO-1 and Bcl-2, and glial cell activation [40]. We found that the expression of both ZO-1 and occludin was downregulated in CIH mice. As ZO-1 and occludin are related to the blood-brain barrier, after the activation of microglia, the blood-brain barrier of hippocampal cells is damaged, leading to the transfer of immune T cells. After Pte treatment, the expression levels of ZO-1 and occludin in the hippocampus significantly increased, suggesting that Pte hindered the progression of immune imbalance and oxidative stress. Therefore, Pte could relieve CIH-induced neuronal injury by regulating the immune imbalance and oxidative stress.

Our study is the first to show that the optimal concentration of Pte in the BV2 cell model was 10 μM. Microglial activation and oxidative stress can increase apoptosis. The apoptosis rate of BV2 cells after LPS + IFN-γ stimulation was confirmed by detecting Bax and Bcl-2 protein levels in the hippocampus and TUNEL staining. In the present study, based on the changes in apoptosis and the increases in p-p38, p-p65, and TRL4 protein levels after Pte intervention, we demonstrated the protective effect of Pte on nerve cells.

## Conclusion

In this study, a CIH model was constructed using C57BL/6 J mice treated with Pte. Activated mouse microglia were then induced with LPS + TNF-γ. We found that Pte protected against CIH-induced neuronal damage by regulating immune imbalance, microglial activation, and oxidative stress. The mechanism of action of Pte is related to the inhibition of p-ERK signal transduction. This study provides a new reference for the treatment of CIH. In a subsequent study, we will further explore the mechanism and relationship of Pte in oxidative stress, inflammation, and immunity.

## Supporting information

S1 Fig Blot/gel image data. (TIF)

S2 Fig Blot/gel image data. (TIF)

S3 Fig Blot/gel image data. (TIF)

S4 Fig Blot/gel image data. (TIF)

S1 File The proportion of Th1, Th2, Th17, and Treg cells in the peripheral blood, analyzed using flow cytometry. (XLS)

S2 File The proportion of Th1, Th2, Th17, and Treg cells in BV2,analyzed using flow cytometry. (XLS)
