# Supplementary figures and images for: Pterostilbene mediates glial and immune responses to alleviate chronic intermittent hypoxia-induced oxidative stress in nerve cells
Source: PLoS One. 2023 Jun 2;18(6):e0286686. doi: 10.1371/journal.pone.0286686 (PMC10237435; doi:10.1371/journal.pone.0286686)

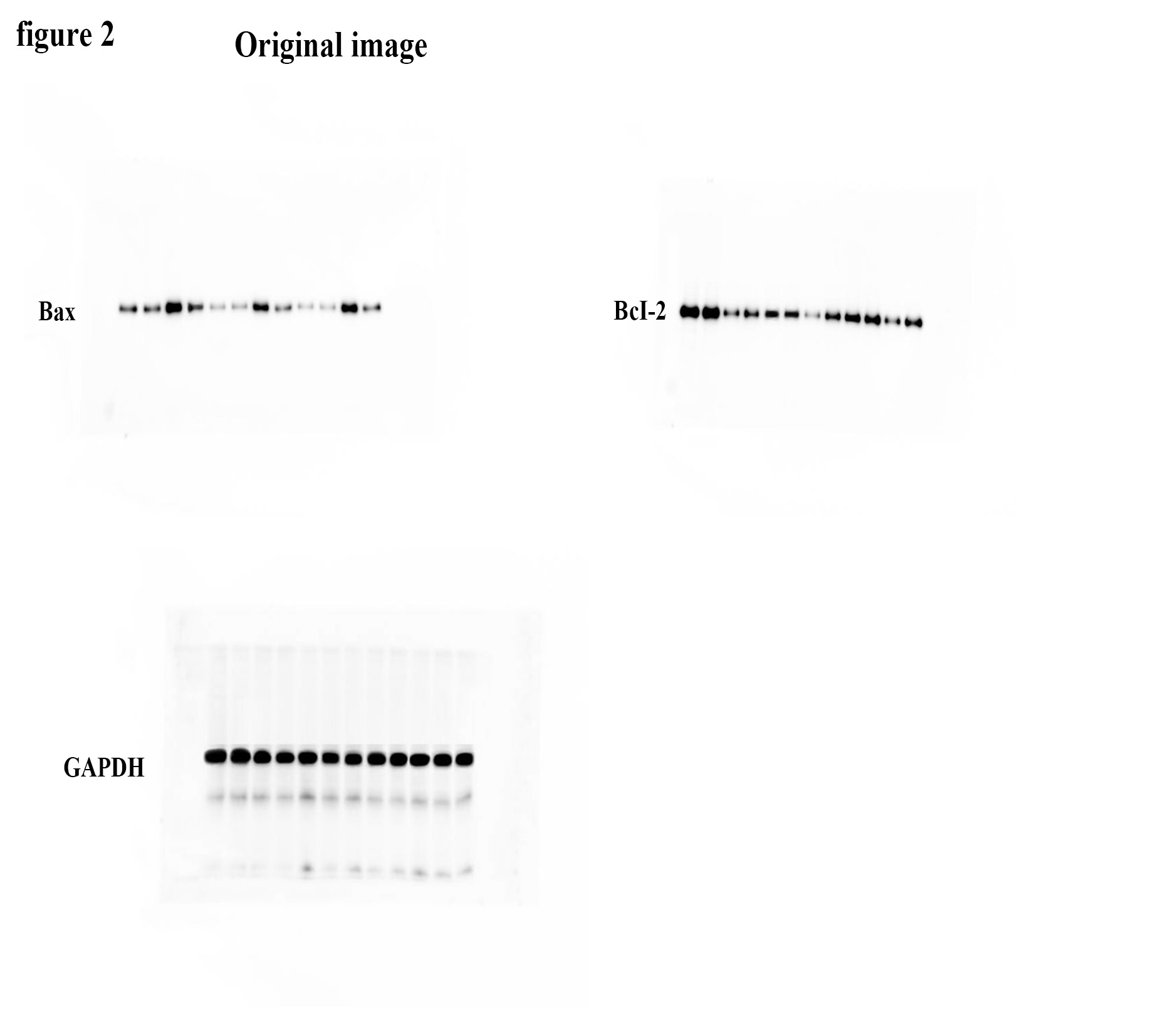

Supplement: S1 Fig — (TIF) [file pone.0286686.s001.tif]

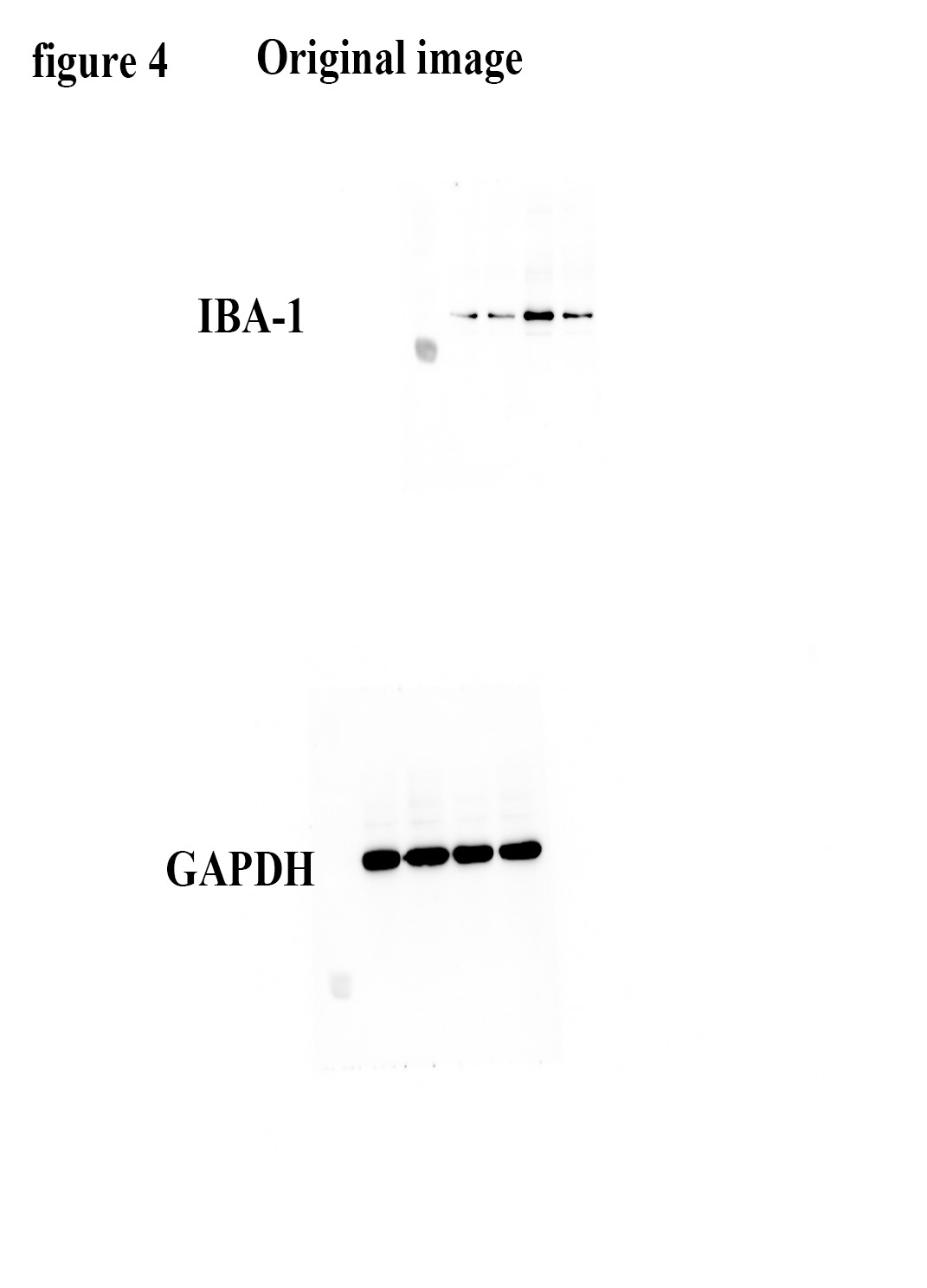

Supplement: S2 Fig — (TIF) [file pone.0286686.s002.tif]

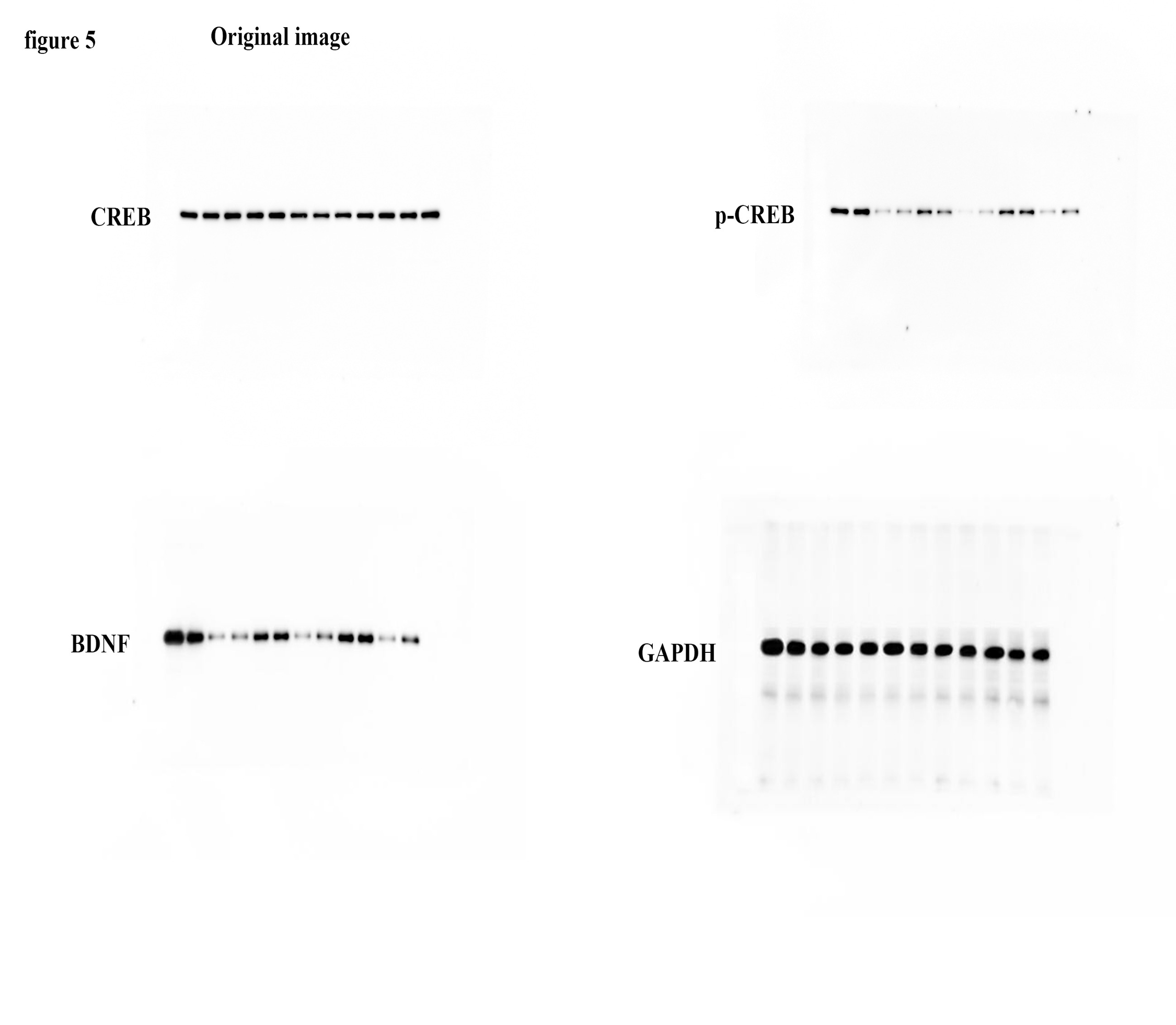

Supplement: S3 Fig — (TIF) [file pone.0286686.s003.tif]

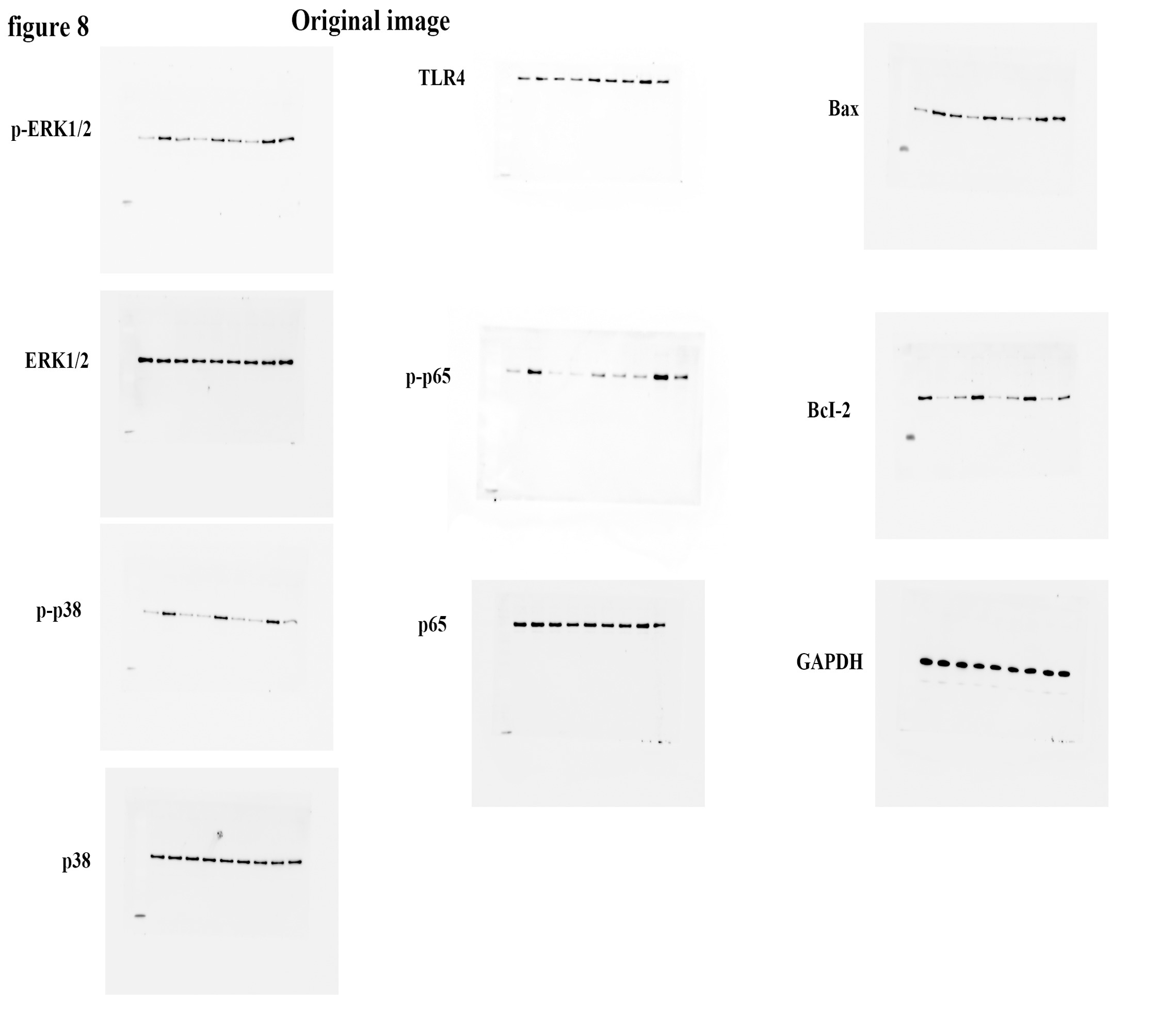

Supplement: S4 Fig — (TIF) [file pone.0286686.s004.tif]
